# Adenoid Cystic Carcinoma Treated With Salvage ZAP-X Radiosurgery in Elderly Patients: A Report on a Case Series

**DOI:** 10.7759/cureus.89388

**Published:** 2025-08-05

**Authors:** Agnieszka Sopel, Monika Rucinska, Marek Derenda, Aneta Lebiedzinska, Sergiusz Nawrocki

**Affiliations:** 1 Department of Oncology, University of Warmia and Mazury in Olsztyn, Olsztyn, POL; 2 Brain, Head and Neck Radiosurgery Center, University Clinical Hospital in Olsztyn, Olsztyn, POL; 3 Department of Neurology and Neurosurgery, University of Warmia and Mazury in Olsztyn, Olsztyn, POL

**Keywords:** adenoid cystic carcinoma, elderly patient, radiosurgery, recurrence locoregional, zap-x® radiosurgery

## Abstract

Management of recurrent adenoid cystic carcinoma (ACC) in elderly patients remains challenging due to comorbidities, functional impairments, and anatomically complex tumor locations that complicate surgical access and increase operative risk. The ZAP-X Gyroscopic Radiosurgery System (ZAP Surgical Systems, Inc., San Carlos, CA, USA) offers a highly precise, non-invasive treatment modality, potentially suitable for salvage therapy in previously irradiated fields and in medically inoperable patients. The article presents two elderly female patients (aged 84 and 89 years) with histologically confirmed recurrent ACC of the nasopharynx and nasal cavity/ethmoid sinus, respectively, who were treated with salvage ZAP-X radiosurgery. The first patient presented with a recurrent lesion measuring 2.1 cm³, while the second patient’s tumor volume was significantly larger at 34.9 cm³, approaching the upper end of published single-fraction volume limits typically deemed manageable for radiosurgical intervention. MRI-based simulation and treatment planning were performed to ensure optimal target coverage and sparing of organs at risk, particularly the optic structures. The first lesion was prescribed 16 Gy to the 52.5% isodose line, while the sinonasal tumor was treated with 10 Gy to the 60% isodose line, both delivered in a single fraction without margins. Treatment plans prioritized steep dose gradients and high conformity indices. Both patients underwent treatment without CTCAE ≥ Grade 2 toxicities. In the first patient, follow-up MRI over 18 months showed significant tumor reduction and sustained clinical improvement. In the second patient, early follow-up showed disease stabilization and symptom palliation. Both patients experienced relief from vertigo, tinnitus, and ocular motility disturbances, respectively, and no new adverse events were noted. WHO performance status remained excellent in both patients. Notably, despite the larger tumor burden in the second patient, effective local control was achieved, demonstrating the potential of ZAP-X-based radiosurgery even in very large-sized lesions. Salvage ZAP-X radiosurgery appears feasible, safe, and effective for the treatment of recurrent ACC in elderly patients, including tumors exceeding the upper size limits traditionally considered for radiosurgical management. The system’s high precision and ability to generate steep dose gradients facilitate safe reirradiation in previously treated, anatomically complex regions. These early findings encourage further prospective studies with larger cohorts and longer follow-up to validate efficacy, monitor late toxicity, and better define the role of radiosurgery in the multidisciplinary management of recurrent head and neck malignancies.

## Introduction

Adenoid cystic carcinoma (ACC) is a rare malignant neoplasm that primarily arises from the secretory epithelial cells of the salivary glands, including both the major and minor salivary glands, and more uncommonly, from the mucosal glands of the nasal cavity, paranasal sinuses, and nasopharynx [1]. Despite its histologically low-grade appearance, ACC is characterized by a paradoxically aggressive clinical behavior marked by perineural invasion, local recurrence, and distant metastases that often occur late in the disease course. The tumor typically exhibits a slow-growing, insidious nature, which often leads to delayed diagnosis [2]. Clinical presentation is usually related to the anatomical site involved and may include nasal obstruction, epistaxis, facial pain, hearing changes, or visual impairment. Given its deep-seated location and close proximity to critical neurovascular structures, complete surgical resection with negative margins can be challenging, especially in recurrent or anatomically complex sites such as the nasal cavity and nasopharynx [3].

Historically, the primary treatment modality for ACC has been surgical excision followed by adjuvant radiotherapy. However, for tumors in surgically inaccessible locations or in patients with contraindications to surgery due to age or comorbidities, radiotherapy plays a central curative role. With the advent of advanced radiation techniques, such as intensity-modulated radiotherapy (IMRT), stereotactic radiosurgery (SRS), and proton beam therapy, the therapeutic index has significantly improved [4]. Among these, ZAP-X (ZAP Surgical Systems, Inc., San Carlos, CA, USA), a novel gyroscopic radiosurgery system, has recently emerged as a highly precise and non-invasive modality that enables safe and effective targeting of intracranial and extracranial lesions without the need for shielding vaults [5]. The unique self-shielding, cobalt-free design of ZAP-X enables high dose conformity and sparing of adjacent normal tissues, making it particularly well-suited for salvage treatment in previously irradiated fields. SRS was selected as salvage therapy for the visible, well-demarcated recurrence, despite the risk of ACC spreading via perineural invasion along cranial nerve pathways, particularly toward the skull base foramina. Evidence for single-fraction SRS in ACC remains limited. The largest retrospective series to date, comprising 55 lesions treated predominantly with Gamma Knife technology, documented a five-year local-control rate of 60% and emphasized the high incidence of marginal failure when SRS is employed as monotherapy [6]. Despite this, local control in recurrent cases was unexpectedly robust, lending support to the use of SRS in extremely elderly and pre-treated patients. Notably, published cases have involved individuals under 80 years of age and tumors with mean volumes of approximately 10 cm³, leaving open questions about the feasibility of SRS in older cohorts and in lesions that exceed these reported dimensions.

This report presents two cases of elderly female patients with ACC located in the upper aerodigestive tract, who underwent salvage radiosurgery using the ZAP-X system following initial treatment failure or recurrence. The gross tumor volume (GTV) was manually delineated on the planning CT images, which had been fused with contrast-enhanced T1-weighted and T2-weighted MRI sequences. The GTV encompassed all contrast-enhancing lesions visible on MRI, as well as asymmetric non-enhancing regions relative to the contralateral anatomy. In accordance with single-fraction SRS protocols and considering the prior high-dose radiotherapy and the proximity of critical organs at risk (OAR), no additional margin was applied. The first patient, aged 84 years, was diagnosed with ACC of the nasopharynx and underwent primary definitive radiotherapy due to medical inoperability. The patient previously declined referral for proton therapy due to the distant location of the treatment center from her place of residence. Despite an initial partial response, local recurrence was identified approximately three years after treatment. Given the anatomical inaccessibility of the recurrent lesion and the patient's desire to avoid surgical intervention, ZAP-X SRS was employed as a salvage treatment strategy. The second patient, aged 88 years, had a long-standing history of ACC of the left nasal cavity and ethmoid sinus, initially treated surgically in 2016. Multiple local recurrences ensued, each managed with repeat resections until the tumor extended toward the orbital structures. Faced with the recommendation for orbital exenteration, the patient opted against further surgery and was referred for salvage radiosurgical treatment with ZAP-X. In both cases, radiosurgery was not only well tolerated but also resulted in symptom stabilization and radiologic improvement.

These cases underscore the challenges associated with the management of ACC in elderly populations, particularly when the tumor is located in areas of complex anatomy or has recurred after multiple interventions. The clinical decision-making process in such patients must balance oncologic control with quality of life considerations and potential treatment morbidity. Salvage options such as ZAP-X radiosurgery, which allow for non-invasive, high-precision intervention, offer a promising therapeutic alternative in this context. While literature on the use of ZAP-X for head and neck malignancies is still emerging, our experience contributes to the growing body of evidence supporting its application in selected cases of recurrent or refractory ACC. The following case series provides a detailed description of clinical presentation, therapeutic approach, and short-term outcomes of ZAP-X radiosurgery in two octogenarian patients with challenging presentations of ACC, highlighting its feasibility, tolerability, and potential efficacy in salvage settings.

## Case presentation

**Figure 1:**
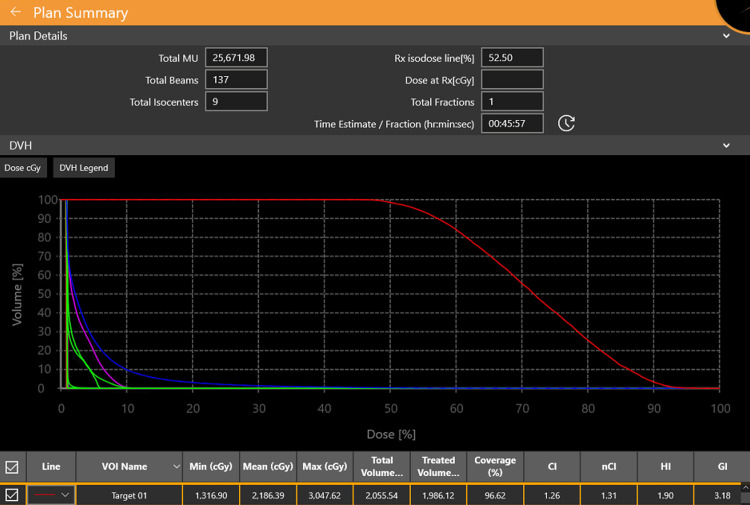
Plan details and histogram of the dose distribution for the first patient.

The first case involves an 84-year-old woman diagnosed in early 2020 with ACC of the nasopharynx (cT1N0M0). She presented with complaints of left-sided aural fullness and progressive hearing loss. Nasal endoscopy revealed a firm, spherical mass occluding the Eustachian tube orifice. CT imaging confirmed a 30 x 20 mm tumor localized to the left nasopharynx, likely originating from the pharyngeal tonsil. Histopathological examination confirmed ACC. Due to the patient’s advanced age and anatomical inaccessibility of the tumor, she was disqualified from surgery during the multidisciplinary tumor board review and referred for radical radiotherapy. The patient underwent definitive IMRT between March and April 2020, receiving a total dose of 69.96 Gy to the primary tumor in 33 fractions of 2.12 Gy each, and 53.71 Gy to the bilateral cervical lymphatic regions (levels II-V) at 1.63 Gy per fraction. She tolerated treatment well with expected toxicities, including Grade 3 mucositis and Grade 2 dermatitis, both managed symptomatically. Following radiotherapy, clinical follow-up indicated gradual improvement, and radiological assessments demonstrated partial regression of the lesion with no new disease. However, over the course of subsequent follow-ups, a slow-growing recurrence was suspected based on patient-reported symptoms of vertigo, left-sided tinnitus, and progressive hearing decline. MRI scans conducted in 2023 identified a recurrent lesion measuring 19 x 17 x 17 mm, infiltrating the torus tubarius and surrounding musculature. The case was re-evaluated by the oncology team, and due to the high-risk anatomical location and patient preference to avoid surgical salvage and proton therapy, she underwent SRS using the ZAP-X platform in September 2023. The total target volume (Target 01) treated with ZAP-X was 2.1 cm³. The prescription dose was 1600 cGy (16 Gy), delivered in a single fraction. The maximum dose within the target reached 3047.62 cGy, highlighting the high-dose gradient achievable with SRS (Table 1). A single fraction of 16 Gy to the 52.5% isodose line was delivered (Figure 1).

**Table 1:** Dose details of organs at risk for the first patient.

Plan Details			
Name	Volume (mm³)	Min dose (cGy)	Max dose (cGy)
Right eye	7403.00	23.84	78.68
Left cochlea	112.00	29.04	32.19
Right lens	69.00	25.16	26.63
Body	6722834.00	3000.00	0.00
Chiasma	640.00	28.61	29.12
Target 01	2056.00	1316.90	3047.62
Right cochlea	100.00	28.14	31.95
Right optic nerve	661.00	28.93	184.18
Left carotid artery	2384.00	28.68	335.76
Left eye	6938.00	24.86	162.24
Left lens	79.00	25.82	27.23
Brainstem	17612.00	27.72	353.73
Left optic nerve	597.00	28.31	29.77

**Figure 2:**
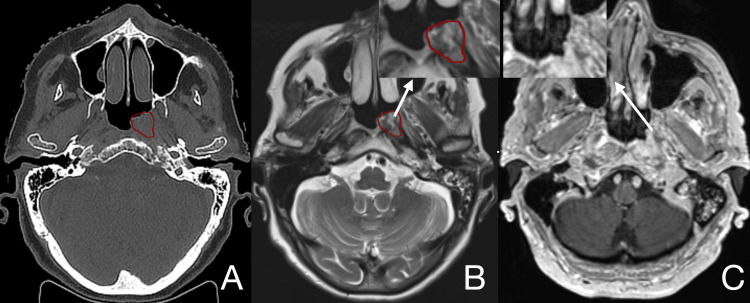
Tumor delineated before radiosurgery in CT (A) and T2 MRI (B), with almost complete regression 18 months after radiosurgery on T1 MRI (C) for the first patient.

The procedure was well tolerated, without acute neurological or otologic complications. At subsequent follow-up, the patient reported symptomatic relief and almost complete regression on MRI imaging (Figure 2).

The second patient, aged 88 years, had a long-standing history of recurrent ACC of the left nasal cavity and ethmoid sinus. Initially diagnosed in 2016, she underwent several surgical resections over the years due to local recurrences in the same anatomical region. Despite repeated operations, the disease persisted, and in mid-2024, she presented with a new recurrence. Clinical findings included left eye visual deterioration, diplopia, and difficulty in ocular movement toward the medial direction. MRI conducted in June 2024 revealed a recurrent mass measuring 37 x 31 x 49 mm with proximity to the orbital structures. While orbital exenteration was proposed as a curative surgical option, the patient declined this approach due to concerns about functional and aesthetic outcomes.

**Figure 3:**
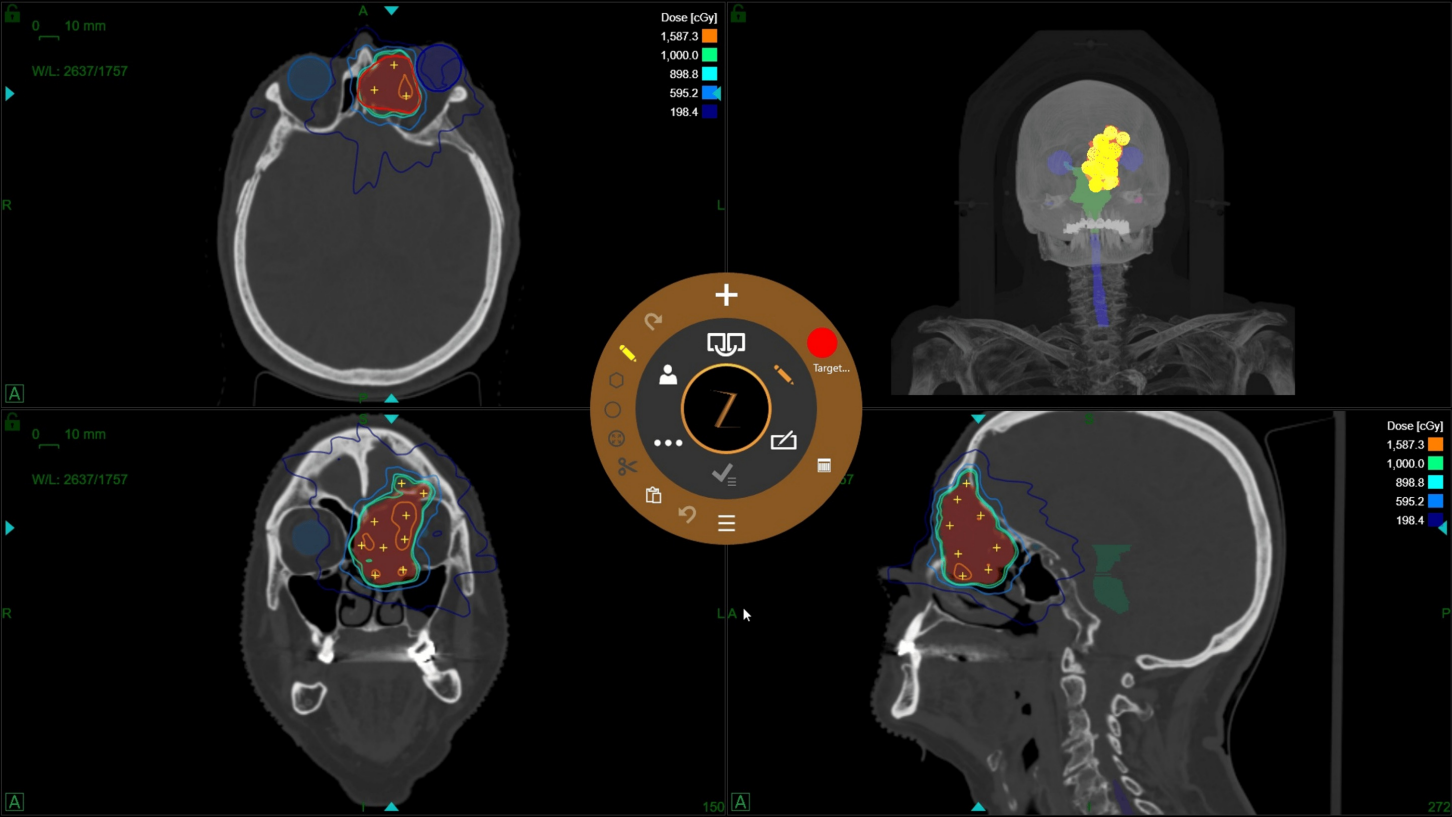
The second patient underwent radiosurgical treatment of a large lesion.

Consequently, she was referred to the radiation oncology team for further management. Given the lesion's recurrence and her age-related surgical risk, SRS using the ZAP-X system was selected as the treatment of choice. The treated target volume was 34,9 cm³. The patient received a prescription dose of 1000 cGy (10 Gy) delivered to the 60% isodose line in a single fraction (Figure 3). The plan prioritized conformality and critical structure avoidance, particularly regarding the adjacent orbit and optic nerve (Table 2).

**Table 2:** Dose details of organs at risk for the second patient.

Name	Volume (mm³)	Min dose (cGy)	Max dose (cGy)
Optic chiasm	347.00	219.79	384.26
Left eye	6246.00	59.67	448.67
Left cochlea	153.00	48.25	82.88
Spinal cord	4778.00	21.78	109.07
Target 01	34941.00	807.94	1984.13
Right optic nerve	560.00	118.40	375.55
Body	9443472.00	3000.00	0.00
Right eye	7105.00	39.95	205.19
Brainstem	24102.00	39.47	237.21
Left optic nerve	417.00	243.55	702.84
Right cochlea	109.00	33.97	37.17

**Figure 4:**
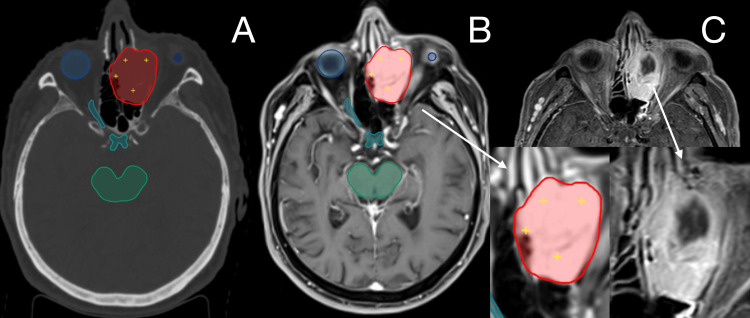
Tumor delineated before radiosurgery in CT (A) and T1 MRI (B), with no progression with central necrosis six months after radiosurgery on T1 MRI (C) for the second patient.

On October 24, 2024, the patient received a single fraction of 10 Gy delivered to the 60% isodose line encompassing the recurrent tumor mass. The procedure was well tolerated. Post-treatment assessments showed stabilization of the disease with radiological improvement in tumor morphology (Figure 4). Notably, the previously observed gelatinous discharge from the eye resolved, ocular movements improved, and there was no further deterioration in visual acuity. Clinical follow-up in December 2024 reported a WHO performance status of 1 and no new complaints. The single-fraction dose of 10 Gy prescribed to the 60% isodose in the second patient was chosen because of the tumor’s size and its proximity to the optic pathways. The lesion measured 34.9 cm³ before SRS and lay against both orbits and the optic chiasm. This volume already exceeded the ≈30 cm³ threshold cited in RTOG 9005 and later consensus guidelines, which advise lowering marginal doses to 12 Gy or less when critical structures must receive no more than 8 Gy to the optic apparatus. 

The most recent MRI performed in June 2025 showed that the mass in the left frontal sinus, anterior and middle ethmoid cells, and maxillary apex had decreased from 37 × 31 × 49 mm to 39 × 22 × 36 mm, an ellipsoid-volume reduction of roughly 45%. The lesion no longer pressed on the medial orbital wall, displayed uniform contrast enhancement, and remained separate from the globe and extraocular muscles. No new bone destruction, cranial-nerve thickening, or nodal enlargement was observed. Taken together, these findings represent a partial radiological response and match the clinical improvement already noted: resolution of diplopia while maintaining a WHO performance status of 1. Actually, the 7.5-month interval shows partial radiological volumetric response. 

Both patients demonstrated favorable tolerance to ZAP-X radiosurgery without any acute toxicities. In the context of advanced age, prior treatments, and refusal or contraindications for extensive surgical procedures, ZAP-X proved to be a feasible and well-accepted salvage modality. These case reports reinforce the potential utility of ZAP-X radiosurgery in managing complex head and neck malignancies such as ACC, particularly in cases of recurrence where traditional interventions may not be safe or desired.

## Discussion

In both cases, ZAP-X radiosurgery offered excellent local control within the short-to-intermediate term, without significant acute toxicity or decline in functional status. Importantly, both patients, aged 84 and 88 years respectively, maintained a WHO performance status of 1 following treatment. This supports the growing consensus that age alone should not be a limiting factor in selecting patients for high-precision radiotherapy. Instead, functional reserve, comorbidities, and patient preferences must guide therapeutic decisions. The patients' refusal or inability to pursue aggressive surgical approaches necessitated a non-invasive alternative that could offer both oncologic efficacy and preservation of quality of life. In this regard, ZAP-X radiosurgery met both objectives.

When comparing these two cases, differences in target volumes and prescribed dose regimens illustrate the adaptability of the ZAP-X radiosurgical platform. The first patient treatment involved a smaller target volume of 2.1 cm³ receiving 1600 cGy to the 52.5% isodose, producing a maximum dose of over 3000 cGy within the lesion. The previous fractionated radiotherapy treatment was performed in a different center and with a different planning system. Therefore, it was not possible to sum the critical doses, and additionally, these doses cannot be summed mathematically due to the different sensitivity of tissues to fractionated radiotherapy compared with stereotactic radiotherapy. In contrast, the sinonasal case involved a larger target of 34.9 cm³ with a prescription of 1000 cGy to the 60% isodose line. Despite differences in prescription, both plans demonstrated strong conformality and safe delivery of ablative doses to anatomically complex regions. This supports the clinical flexibility of ZAP-X for tailoring treatment regimens based on tumor size, location, and patient-specific constraints. While published data on ZAP-X in head and neck malignancies remain sparse, emerging reports indicate its efficacy in small, well-defined, and recurrent lesions located in critical regions. The largest series of patients with ACC treated with salvage radiosurgery reported 17 cases with similar doses; however, our patients were older, and our second patient had an exceptionally large tumor treated [6]. The ability to deliver stereotactic doses with tight margins while sparing adjacent OAR is especially relevant in reirradiation scenarios. Compared to proton therapy, ZAP-X may offer logistical advantages and similar dosimetric profiles in small-volume lesions. A 2023 UK benchmark that replanned identical cases on six current stereotactic-radiosurgery systems (Gamma Knife Icon, CyberKnife S7, Brainlab Elements on Elekta linacs, Brainlab Elements on Varian linacs, Varian HyperArc, and Zap-X) found virtually identical plan quality: mean target coverage, 98.2%-99.7%; Paddick conformity, 0.722-0.894; and gradient index, 3.52-5.08. Trade-offs were minor (CyberKnife: highest conformity, lower-energy; Gamma Knife Icon/Zap-X: steepest fall-off) and did not reach statistical or clinical significance. Compared with the 2016 benchmark, interplatform gaps have narrowed, supporting the conclusion that no contemporary SRS device offers a meaningful dosimetric advantage over the others [7]. Further comparative studies are needed to establish its place in the treatment hierarchy. Additionally, long-term follow-up is essential to assess late toxicity, local control, and disease-free survival in this unique patient subset. 

These two cases have important limitations. In the first patient, we have used radiosurgery after fractionated radical radiotherapy. Re-irradiation in head and neck tumors is better understood for fractionated radiotherapy than for the combination of fractionated radiotherapy with radiosurgery. In our patient, cumulative dose and biological effective dose summation for re-irradiation both for the tumor and critical organs have not been possible for two reasons. Firstly, the initial fractionated radiotherapy was performed at a different institution using a different planning system so that we have had available limited parameters of previous treatment. We considered maximum doses in OARs (optic chiasm received 4.3 Gy, brainstem received 36.2 Gy, and doses in other organs were insignificant). Secondly, summation of doses from conventional fractionated radiotherapy and SRS is clinically unreliable, as the linear-quadratic model used in fractionated radiotherapy underestimates the biological effects observed in radiosurgery. Consequently, treatment planning for ZAP-X in this case was based primarily on radiosurgical OAR constraints and clinical judgment. In the second case, we treated the patient after several surgical interventions for the unusually large target volume for radiosurgery, so that the prescribed doses selected were chosen arbitrarily based on very limited data published. Another limitation of our report is the relatively short follow-up. Finally, this report describes only two patients from a single institution, limiting generalizability.

In summary, our experience with these two cases demonstrates that ZAP-X radiosurgery is a feasible, well-tolerated, and potentially effective salvage option for recurrent or unresectable ACC of the head and neck. It should be considered in carefully selected patients, particularly when traditional treatments are no longer viable. The role of ZAP-X in ACC management warrants further investigation through prospective trials and multi-institutional registries to better define its indications and outcomes.

## Conclusions

ZAP-X SRS was successfully used as a salvage treatment in two elderly patients, aged 84 and 88 years, with recurrent ACC of the upper aerodigestive tract. Both patients had a long history of prior interventions, including radiotherapy for the first patient and multiple surgeries for the second patient. Despite advanced age and comorbidities, the treatment was well tolerated and resulted in symptom control, radiologic improvement, and preservation of functional status (WHO performance status of 1). Notably, in one case, the target volume exceeded the conventional upper limits for SRS, yet treatment was delivered effectively without significant toxicity. These findings underscore the potential of ZAP-X to safely address tumors in surgically challenging and previously irradiated locations, even when traditional size criteria are exceeded. This experience supports the inclusion of ZAP-X in the therapeutic arsenal for recurrent ACC, particularly in geriatric patients or those unfit for surgery. Further studies are needed to refine its indications and long-term outcomes.

## References

[1] Coca-Pelaz A, Rodrigo JP, Bradley PJ (2015). Adenoid cystic carcinoma of the head and neck--an update. Oral Oncol.

[2] Cantù G (2021). Adenoid cystic carcinoma. An indolent but aggressive tumour. Part B: treatment and prognosis. Acta Otorhinolaryngol Ital.

[3] Garden AS, Weber RS, Morrison WH (1995). The influence of positive margins and nerve invasion in adenoid cystic carcinoma of the head and neck treated with surgery and radiation. Int J Radiat Oncol Biol Phys.

[4] Atallah S, Marc M, Schernberg A, Huguet F, Wagner I, Mäkitie A, Baujat B (2022). Beyond surgical treatment in adenoid cystic carcinoma of the head and neck: a literature review. Cancer Manag Res.

[5] Ukoha CD Jr, Nguyen N (2021). Pulmonary mucormycosis: an interesting case of Rhizopus mucormycosis. Cureus.

[6] Hong S, Garces YI, Price KA, Shinya Y, Parney IF, Link MJ, Pollock BE (2024). Treatment outcomes of single-fraction stereotactic radiosurgery for adenoid cystic carcinoma: a case series of 55 patients. J Neurooncol.

[7] Paddick I, Mott J, Bedford J (2023). Benchmarking tests of contemporary SRS platforms: have technological developments resulted in improved treatment plan quality?. Pract Radiat Oncol.

